# Wood Densitometry in 17^th^ and 18^th^ Century Dutch, German, Austrian and French Violins, Compared to Classical Cremonese and Modern Violins

**DOI:** 10.1371/journal.pone.0046629

**Published:** 2012-10-10

**Authors:** Berend C. Stoel, Terry M. Borman, Ronald de Jongh

**Affiliations:** 1 Division of Image Processing, Department of Radiology, Leiden University Medical Center, Leiden, The Netherlands; 2 Borman Violins, Fayetteville, Arkansas, United States of America; 3 Cremonese Vioolbouw Amsterdam, Amsterdam, The Netherlands; The Pennsylvania State University, United States of America

## Abstract

Classical violins produced by makers such as Antonio Stradivari and Guarneri del Gesu have long been considered the epitome of the luthier's art and the expressive tool of choice for the most celebrated violinists. It has been speculated these makers had access to wood that was unique in some way and that this was responsible for their acclaimed tonal characteristics. In an attempt to discern whether the above conjecture is true, we analyzed 17 modern and classical Dutch, German, Austrian and French violins by wood densitometry using computed tomography and correlated these results with our previous study of modern and Cremonese violins; in all studying 30 instruments of the violin family. In order to make this comparison possible we developed methods to cross calibrate results from different CT manufacturers using calibration wood pieces. We found no significant differences in median densities between modern and classical violins, or between classical violins from different origins. These results suggest that it is unlikely classical Cremonese makers had access to wood with significantly different wood density characteristics than that available to contemporaneous or modern makers.

## Introduction

Instruments of the violin family produce sound by creating perturbations of the air through movement of the top and back plates of the instrument as well as movements of the instrument body as a whole. The efficiency and aural characteristics of such a system are dependent on its design, the mechanical properties of the individual parts and their interactions [1]–[4]. Violins made exactly alike but with different woods would sound quite different, highlighting the importance of the mechanical properties of the materials used. There are many properties that play a role in wood behavior, although the key triumvirate consists of density, Young's modulus and damping. Knowledge of the density of the materials used in the construction of violins sheds light on how an instrument performs and why. Wood density varies greatly within trees for numerous reasons [5]–[10], as well as from external causes such as aging and treatment of the wood (either intentional or not) [11].

In a previous study [12], computed tomographic (CT) densitometry was used to analyze the density of woods used by the classical Cremonese violin makers Antonio Stradivari and Giuseppe Guarneri del Gesu. The results were then compared to values obtained from modern instruments. The median density of the woods did not differ significantly, but the variations in density between early and late growth (density differential) were less pronounced in the antique Cremonese violins. Additionally, the antique Cremonese wood showed considerably less variation in median density and density differential as opposed to modern violins which showed more variation in these parameters.

In that study it could not be tested whether these differences between antique and modern violins were due to aging, treatment, or other influences. It will be difficult if not impossible to ever test these hypotheses definitively. However, if this was indeed the case one could hypothesize that these alterations to original material may not have been equally applied throughout 17th and 18th century Europe and was possibly restricted to classical Cremona only. Chemical treatments [13]–[15] and biological treatments such as ponding [16] are known to reduce density and these factors, could potentially account for density differences between countries of origin beyond those expected strictly due to geo- and topographical variations. In addition, if the differences between the antique Cremonese and modern violins found in the previous study could be reproduced by a study with violins of the same age but from different European countries, this would provide additional insight into the likelihood of the aging hypothesis.

**Figure 1:**
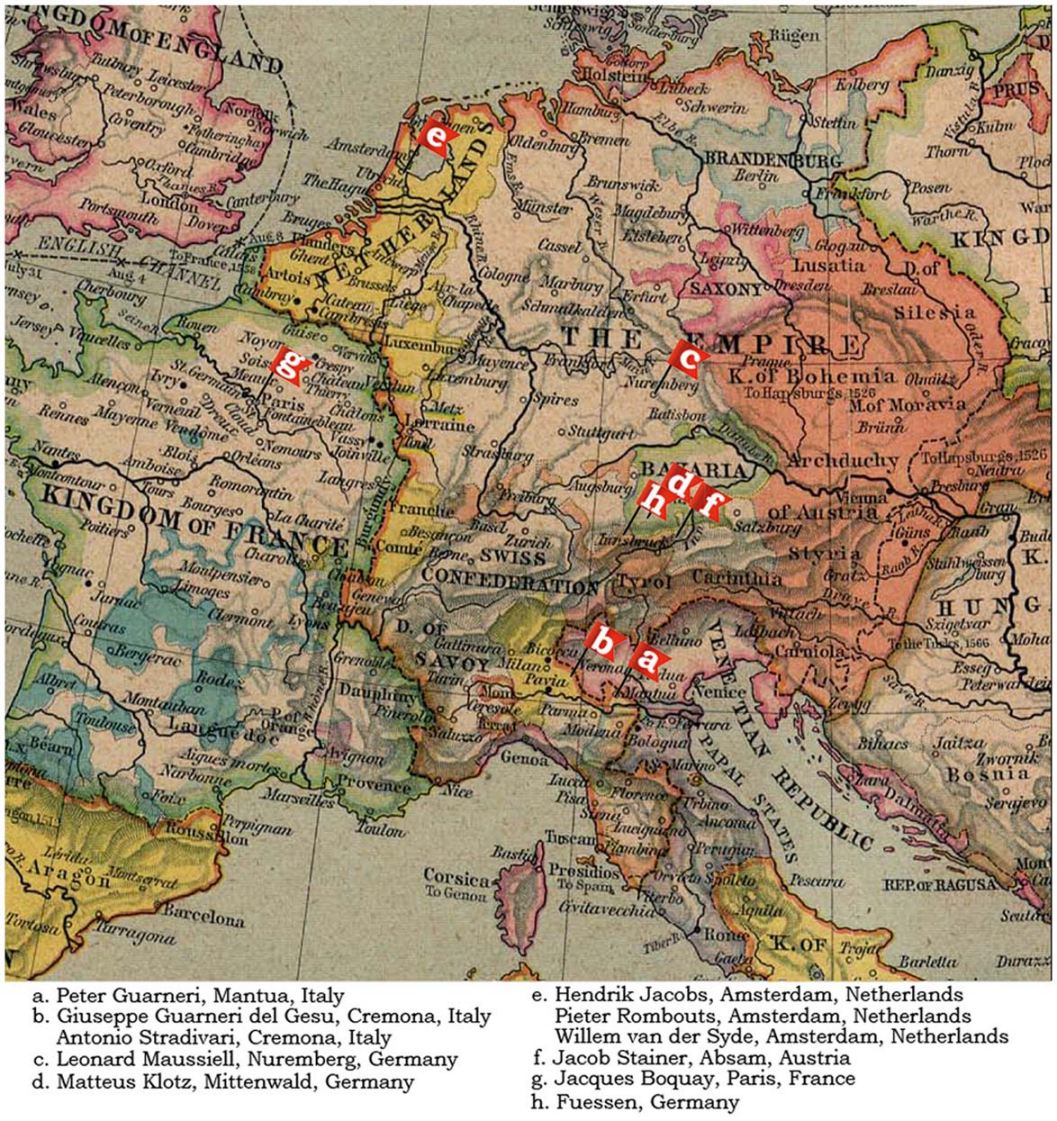
Geographical distribution of the antique instruments studied.

To this end, we applied CT wood densitometry to study the differences in median density and density differential between modern violins and antique violins from the Netherlands, Germany, Austria and France. The approximate geographical distribution of the makers is presented in Figure 1. The Austrian and German violins originated in close proximity and will be considered as one group. We then compared these results with the results from the previous study on Cremonese violins.

In order to make this comparison possible, we developed a normalization procedure to compensate for the differences between CT scanners used in both studies. This procedure uses a calibration wood-set that was scanned immediately prior to scanning the instruments and each wood sample was then analyzed at the same location to ensure accuracy.

## Methods

For our purposes “antique” was defined as an instrument built before 1750 and “modern” as one built within the past 50 years. Further, the antique instruments needed to have certificates of authenticity or of timeframe and region/country of origin. The antique instruments, separated by country of origin included six Dutch, one French, one Italian, five German and Austrian violins, and from the former study five Italian Cremonese violins (Tables 1 and 2).

**Table 1:** Instruments in the current study.

Category	Label	Maker	Date	Origin
Antique	1. PGM1698	Peter Guarneri of Mantua	1698	Mantua, Italy
	2. HJ1685	Hendrik Jacobs	∼1685	Amsterdam, NL
	3. HJ1694	Hendrik Jacobs	1694	Amsterdam, NL
	4. PR1722	Pieter Rombouts	1722	Amsterdam, NL
	5. PR1710	Pieter Rombouts	∼1710	Amsterdam, NL
	6. WS1695	Willem van der Syde	1695	Amsterdam, NL
	7. WS1690	Willem van der Syde	1690	Amsterdam, NL
	8. LM1728	Leonard Maussiell	1728	Nuremberg, Germany
	9. F1720	NN	1720	Füssen, Germany
	10. MK1700	Matteus Klotz	1700	Mittenwald, Germany
	11. JS1655_1	Jacob Stainer	∼1655	Absam, Austria
	12. JS1755_2	Jacob Stainer	∼1655	Absam, Austria
	13. JB1719	Jacques Boquay	1719	Paris, France
Modern	A. TB2005	Terry Borman	2005	Salt Lake City, USA
	B. TB2007	Terry Borman	2007	Fayetteville, USA
	C. RJ2008	Ronald de Jongh	2008	Amsterdam, NL
	D. NN1960	Unknown	∼1960	Germany

All were violins, except PR1710, viola.

Note: Due to changes in political boundaries, “origin” in our usage denotes the current geopolitical boundary of the city of origin. NL: the Netherlands, USA: United States of America.

**Table 2:** Instruments in the former study.

Category	Maker	Date	Origin
Antique	Giuseppe Guarneri del Gesu	1734	Cremona, Italy
	Giuseppe Guarneri del Gesu	1735	Cremona, Italy
	Giuseppe Guarneri del Gesu	1735	Cremona, Italy
	Antonio Stradivari	1715	Cremona, Italy
	Antonio Stradivari	1734	Cremona, Italy
Modern	Terry Borman	1995	Salt Lake City, USA
	Terry Borman	2005	Salt Lake City, USA
	Terry Borman	2005	Salt Lake City, USA
	Terry Borman	2006	Fayetteville, USA
	Alvin Thomas King	2000	Potomac, USA
	Alvin Thomas King	2000	Potomac, USA
	Guy Rabut	2000	New York City, USA
	Guy Rabut	2000	New York City, USA

All were violins, except Borman1995, viola.

For this study all violins were scanned on a Toshiba Aquilion 64 CT scanner at the Leiden University Medical Center using a standardized image acquisition protocol. This protocol was optimized to facilitate a translation from the current results to those of the prior study in New York, where a Siemens 64 Cardiac scanner from Mount Sinai hospital was used.

### Protocol optimization

**Figure 2:**
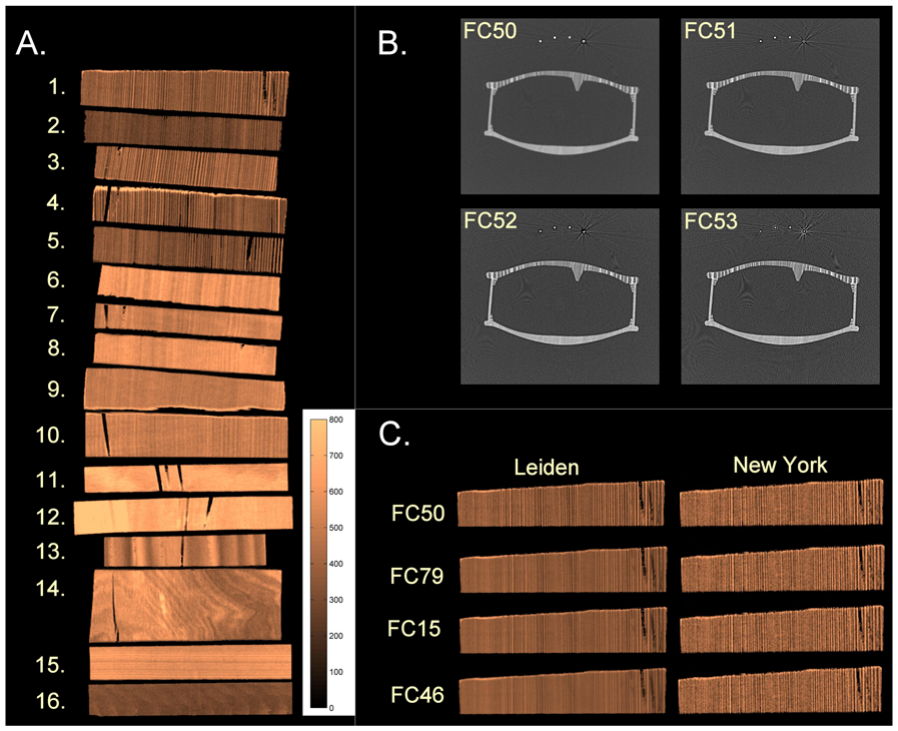
Protocol optimization. **a.** Calibration wood-set composed of different wood sample pieces; #'s 1–5,16, picea abies, #'s 6–10,15, acer pseudoplatanus, #11 fagus grandifolia, #12 acer glabrum, #13 populus albus, and # 14 juglans nigra. **b.** Comparing differences in metal artifacts from the metal strings in a CT scan of a violin; **c.** Images of the first calibration wood piece, obtained from the four candidate filters.

The calibration objects (Figure 2a) consisted of six samples of Picea abies (Norway spruce), six of Acer pseudoplatanus (Eurasian maple), one of Fagus grandifolia (American beech), one of Acer glabrum (rocky mountain or rock maple), one of Populus alba (silver poplar), and one of Juglans nigra (eastern black walnut). The Picea abies and Acer pseudoplatanus samples are of tone-wood quality for the tops and backs of violins, respectively. These were scanned and images were reconstructed using every possible reconstruction filter on the Toshiba scanner. To obtain the optimum correlation, median density, density differential (see Image Analysis) and metal artifacts should match those of the Siemens scanner. This was found by first assessing visually the density, contrast, noise and overshoot effects, focusing on the spruce wood piece at the top of the stack in Figure 2a, producing in total 11 candidate reconstruction filters. Since many different image quality aspects were considered which could not currently be assessed quantitatively, this optimization was carried out visually. The candidate filters were evaluated further in a second step, by applying these filters to the CT scan of one violin. The Moiré-like artifacts (Figure 2b) from the metal strings were then compared between filters, yielding four final candidates (Figure 2c). The final choice was made by visually evaluating the images of the calibration wood pieces obtained from these four candidate filters.

The final image acquisition protocol was as follows: 80 kVp, 50 mA, rotation time 1 sec, collimation 64×0.5 mm, with a pitch factor 0.25, scanned field of view of 320 mm, 250 mm reconstruction diameter for all instruments, slice thickness 0.5 mm with an increment of 0.3 mm and reconstruction filter FC50.

**Figure 3:**
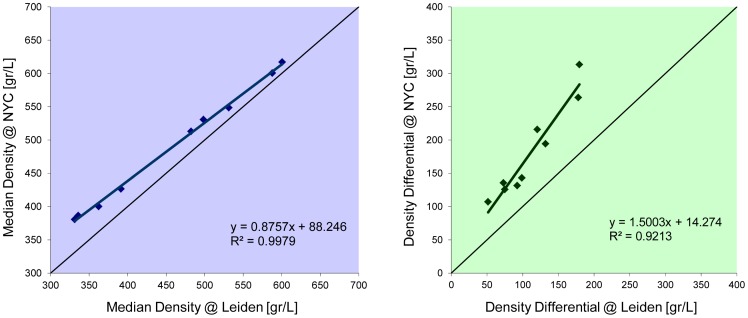
Correlation of density measurements between the CT scanners of Leiden and New York City (NYC).

Since locally wood density can vary considerably, a computer program was developed using image registration that matches regions of interest automatically between two images of the same piece of wood guaranteeing the identical sample area was used each time. During image registration, rigid transformations were only used, without any interpolation to prevent any effects of interpolation on image characteristics. The mean squared difference was used as similarity metric. After manual initialization, this metric was optimized in an area of +/−5 pixels in x- and y-direction. After matching, the translation between the two CT scanners was determined by linear regression between the two measurements of median density and density differential (Figure 3a, 3b). In other words, the regression equations in Figure 3 were used as transfer functions between the two scanners. The small residual errors and high correlation coefficients indicate that the translation was reliable.

### Image Analysis

**Figure 4:**
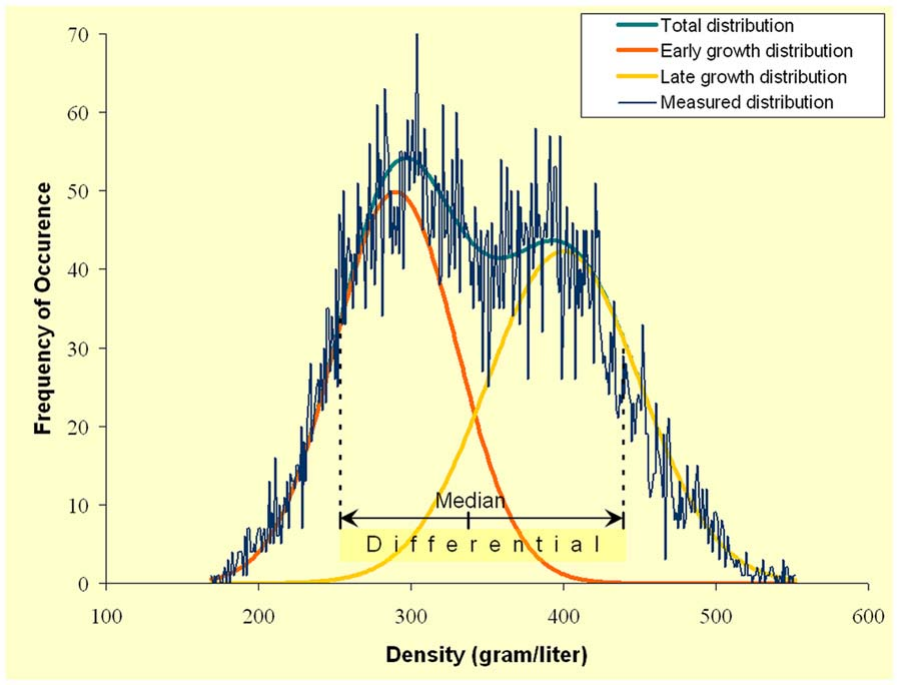
Example of a distribution of densities in a region of interest in a top plate, from which the median value and differential is calculated.

After protocol optimization, the images from the violins were analyzed automatically with in-house designed software [12] based on MatLab (The MathWorks inc., version R2007a). This produces 2-dimensional images (‘density maps’) of the top and back plates, in which each pixel represents the local density in gram/liter (or in kg/m^3^ with the same magnitude). In accordance with our previous study, measurements were taken from the top and back plates at five different regions of interests (ROI) covering an area of 3×3 cm^2^: two in the upper bout (one each on the bass and treble sides), the same for the lower bout, and one at the center of the plate adjacent to the center seam (not including the glue line). Care was taken to not include any regions with repair patches or imaging artifacts. In Figure 4 this methodology is illustrated, where an example is given of a distribution of the frequency of occurrence of densities in a region of interest in a top plate (blue graph). The orange and yellow Gaussian curves indicate the underlying separate distributions of the early and late growth grains, respectively. These combined distributions result in the total distribution (in dark cyan) that resembles the measured distribution. The differential is defined as the difference between the 90^th^ and 10^th^ percentile point, which is a measure of the difference in density between the two separate distributions of early and late growth. The median value represents the density value that splits the total distribution in half, as an alternative to the average value. The final density value for each violin was the average of the measurements from individual ROIs. Since plate thickness affects apparent density values [12], the resulting density values were corrected for this influence by using a separate calibration wood-set. This second calibration set was composed of four 10 cm×4 cm samples each of Picea abies and Acer pseudoplatanus. Each species sample was cut from adjacent wood sections in varying thicknesses of 2 mm, 2.5 mm, 3 mm and 4.5 mm. These results were then calibrated to match the New York CT scanner using the relation in Figure 3, based on the first calibration set.

### Density Differences between Early and Late Growth

Clear consensus does not exist on the definition of the early/late growth transition point; several models have been proposed over the years. The definition of latewood in softwoods was described by Mork [17] (Mork's Index) in 1928 and has been widely accepted. He describes a relationship between the adjacent cell walls of two tracheids being of equal or greater thickness than half the radial diameter of the adjoining tracheid's lumens. Those not falling within these guidelines would be considered early growth. Other, more recent methods using X-ray densitometry include the “Fixed Density Transition” method using a fixed density as the transition point [18], the “Min/Max” method based on the relationship between high/low readings [19], the “Maximum Derivative” method based on the maximum of the derivative function describing intra-ring density variations, and the “Inflexion Point” method [20] based on spline functions. We used the method outlined in Stoel et al [12], in which this transition was defined intrinsically by measuring the difference between the 90^th^ and 10^th^ percentile density values of the histogram generated from each ROI. Using each of the above methods, one would arrive at slightly different results.

### Influence of Grain Line Width on Wood Densitometry

Medical CT scanners have a minimum (nominal) pixel size of 0.5×0.5×0.5 mm with a limited resolution of the resultant images. Therefore it may not depict accurately all grain lines (also known as “growth ring” or “growth increment”). Due to this constraint grain line frequency above approximately 8 grain lines p/cm could not be adequately resolved, potentially influencing the median density and density differential measurements. In order to quantify this influence, we conducted two tests.

**Figure 5:**
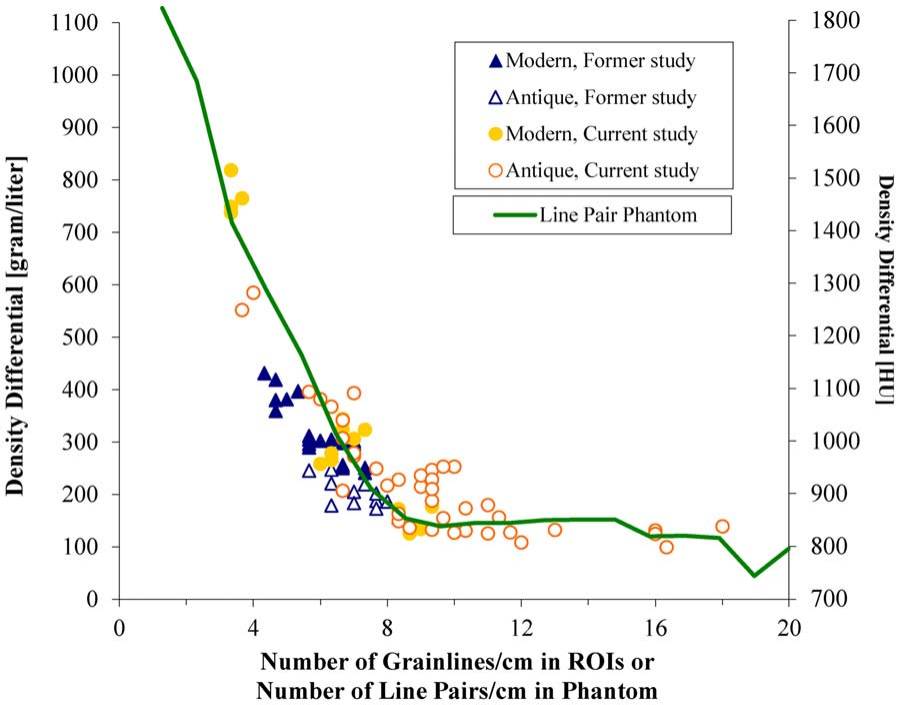
Correlation between the number of grain lines/cm in spruce tops and the apparent density differential; Resolution/contrast test by the Catphan® phantom showing a similar shape.

First, we determined the number of grain lines in the measured areas from high resolution photographs of the violins. To do this we scaled and overlaid the density maps with indicated ROIs to the photographs of the top plates of the violins. We then manually counted the number of grain lines in the four lateral ROIs that were used for taking density samples and calculated the number of grain lines per centimeter. Central ROIs were not included because there were too many missing values in counting grain lines due to the thicker varnish layer, superficial damage in these regions and rosin buildup clouding the view. As the grain lines become narrower the scanner has increasing difficulty differentiating between early and late growth causing a drop in apparent differential (Figure 5).

In a second experiment, we verified this relation, using the line pair section from a Catphan ®500 phantom (The Phantom Laboratory), and scanned with the same imaging protocol. This phantom consists of 21 areas of line pairs, made of aluminum sheets alternated by gaps of epoxy. The number of line pairs varied from 1 to 21 lp/cm, with a gap size from 0.5 cm to 0.024 cm. The CT images of the phantom were analyzed in the same way as for the violins, by calculating the median density and density differential in each ROI covering the line pairs.

The density differential for the violins was plotted against the number of grain lines per centimeter in the lateral ROIs, for which we were able to count them (n = 91), see blue and yellow markers in Figure 5. The green curve indicates a similar relation found in the line pair phantom, in which the density differential measure (in Hounsfield Units on the right y-axis) is plotted against the number of line pairs. Because of the higher contrast between epoxy and aluminum material in the phantom, density differentials are much higher (see the y-axis on the right side of the graph).

We did not find a correlation between median density and grain line width. Therefore, the median density readings were not affected by this limitation in CT resolution.

At a later date it may be possible to correct the differential measurement for this relation, although probably only for frequencies lower than 8 grain lines per cm. For our current purposes we focused our analysis predominantly on the median densities. In our former study this confounder did not affect our results because the grain line width was similar between the modern and Cremonese violins (Figure 5) as well as substantially wider in general.

## Results

**Figure 6:**
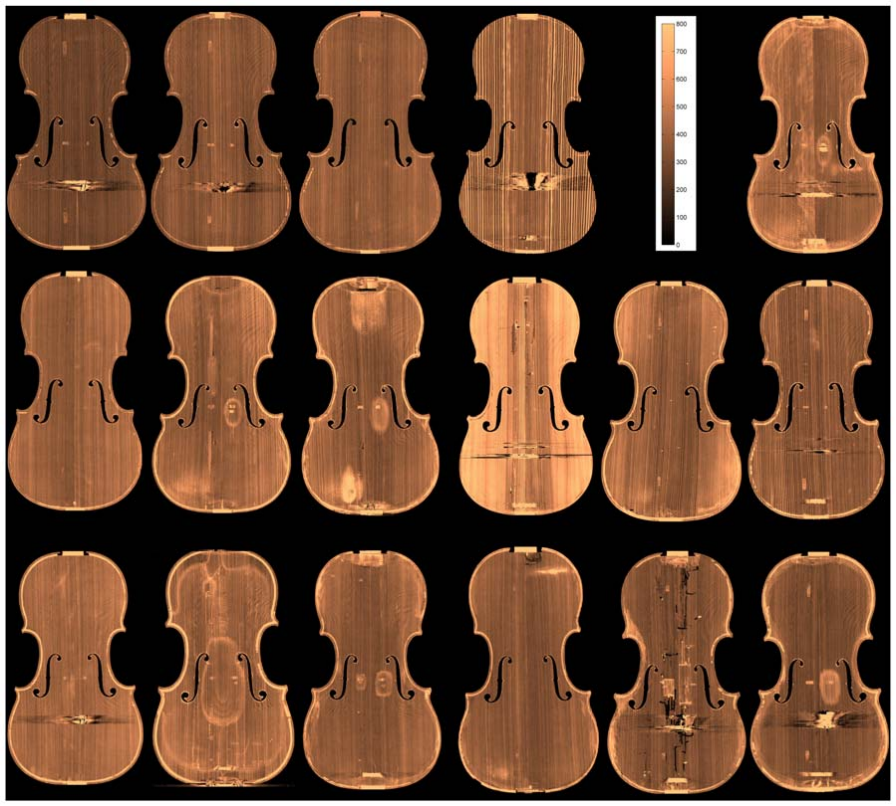
Density maps in gram/liter of the top plates.

**Figure 7:**
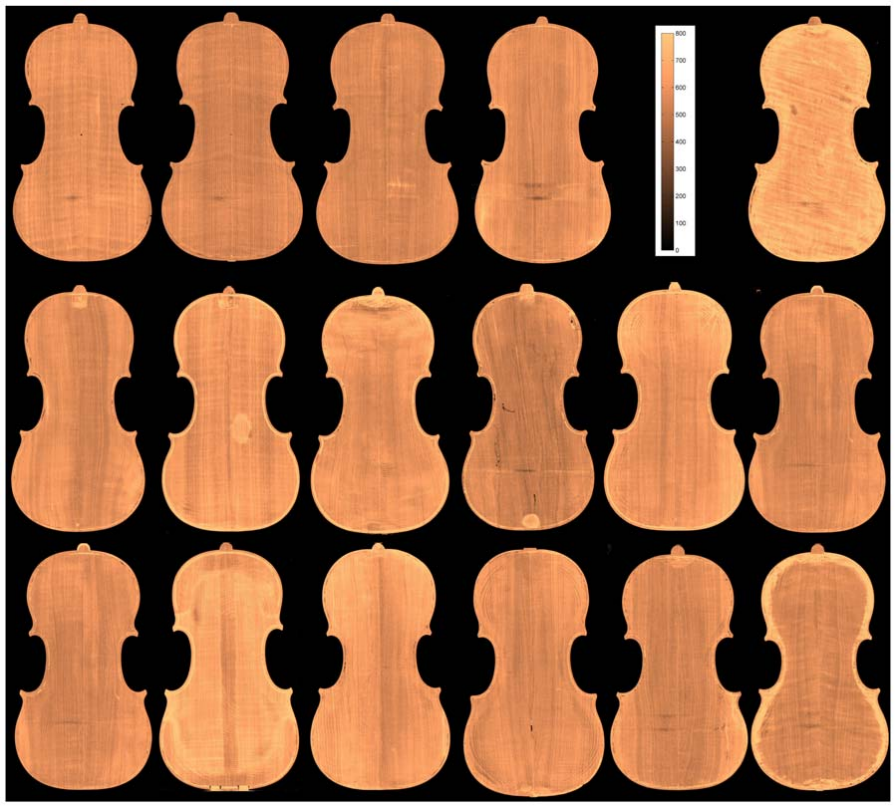
Density maps in gram/liter of the back plates.

The density maps of the top and back plates are displayed in Figure 6 and 7, respectively. In the top row, the four modern violins and the scale are displayed. The remaining images are from antique violins.

### Comparison between Antique and Modern instruments

**Figure 8:**
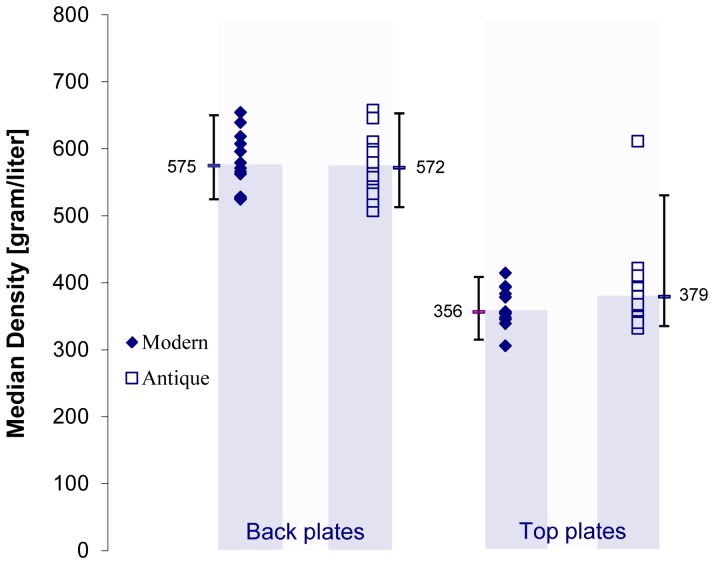
Comparison of the median density between modern and antique back and top plates from this study. Error bars indicate median values and the 95% confidence intervals.

The maple wood of the backs have a median density of 572 g/L for the antique vs. 575 g/L for the modern (Figure 8), which is a surprisingly small difference (95% Confidence Interval, CI: [513–653] g/L and [525–650] g/L; T-test, p = 0.56). The spruce wood of the tops of the antique instruments is slightly denser than that of the modern instruments (Figure 8): median density 379 g/L vs. 356 g/L (CI: [335–531] and [315–409], p = 0.16), which is different from our previous work. There was one antique top plate with an extremely high density, comparable to that of maple.

### Comparison between Countries of Origin

**Figure 9:**
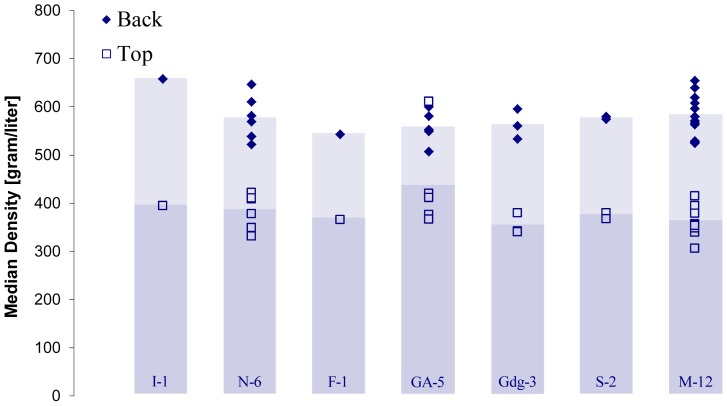
Graph comparing the median density by country of the back and top plates and summed modern instruments. Country-Quantity: I-1, Italy (other than Cremona); N-6, the Netherlands; F-1, France; GA-5, Germany/Austria; Gdg-3, Italy (Guarneri del Gesu); S-2 Italy (Stradivari); M-12, Modern instruments.

For comparing density values of the violins from different countries of origin, we pooled the data from the former and current studies using the calibration technique described in the methods section. Figure 9 displays the median density of the top and back plates of the instruments separated by country of origin: Italy other than Cremona (I-1), Netherlands (N-6), France (F-1), Germany/Austria (GA-5), along with data from the Guarneri de Gesu (Gdg-3), Stradivari (S-2) and modern violins (M-12) from the previous and current study. The bars in the figure represent the mean value of the measurements.

The maple back plates all have median densities fairly similar to one another regardless of country of origin with the I-1 violin being the densest. This may have been due to technical difficulties related to its condition. The median density of the spruce of the German/Austrian top plates was skewed by the density readings of one violin, which were even higher than most of the backs in this study.

## Discussion

Classical violins produced by makers such as Antonio Stradivari and Guarneri del Gesu have long been considered the epitome of the luthier's art and the expressive tool of choice for the most celebrated violinists. Hence, these classical Cremonese violins are thought to be superior to two groups of violins: a) modern violins (potentially due to their difference in age/ageing, origin of the wood, design or wood treatment); b) other antique violins from the same time period (potentially due to differences in origin, design, or wood treatment). The goal of this study was to improve our insight into the material properties of these different groups of violins using CT densitometry.

Density is one of the key material properties contributing to sound production, and could therefore partly explain these acoustical differences. In making musical instruments, there are many material properties that effect how wood, which is an anisotropic material, performs acoustically. For an instrument maker the three key characteristics are density (ρ), stiffness (E) (Young's elastic modulus along and across the grain, and in shear), and internal damping (Q), along and across the grain. The relationship between density and Young's modulus [21] along the grain is well established and first-order approximations of this relationship are readily apparent as presented in graphical format by Ashby et al [22]. This was further quantified specifically in regards to musical instruments by Detloff [23] who found a statistically significant (r = 0.78) linear correlation. It has also been shown [24] that there is a linear relationship between E/ρ and Q. Density is a key determinant of the triad of wood properties that most impact sound characteristics and having reliable information regarding density can therefore provide a glimpse into values of the other two significant material properties.

In this study we compared Cremonese violins and violins of differing origin from the same time period, thereby eliminating one variable (i.e. age/ageing). Since we found no significant differences in median density between antique violins of Cremonese vs. other origins and modern instruments, this indicates that Stradivari and Guarneri del Gesu did not choose, or have access to, wood significantly different in density than contemporaneous or modern makers.

Our results show only small differences in the densities of the maple woods chosen by instrument makers for the backs of their instruments from several countries and over a time-frame of over 300 years. Wood chosen by modern makers is quite similar. The median densities of the spruce tops chosen by modern makers (median value, n = 12∶356 g/L) were on average lower than those chosen by antique makers (median value, n = 18∶379 g/L). This difference is not statistically significant and could therefore be attributed to chance. Figure 8 graphs median density of the instruments in this study. The top of one violin was so remote from normal that it is probably what is referred to as ‘reaction wood’ and would not be chosen by meticulous makers; either from years of empirical experience, or on a biological basis due to the redistribution of endogenous auxins, which alter lignin content creating thicker cell walls, higher density, shorter tracheids, adverse changes in all three layers of the cell walls particularly S2; all leading to reduced speed of sound and radiation ratio [25], [26]. Apart from this one instrument only small differences in median spruce density were found. Spruce wood is made up of approximately 42% cellulose, 27% hemicellulose, 28% [5], [27], [28]. Lignin is the most stable of the main components [29], [30] with both cellulose and hemicellulose losing ∼5–10% over time. Early wood has higher concentrations of lignin due to the cell wall make-up. With the more stable component being the early wood, this leaves changes in the late wood to account for the reduced density differentials found in our previous study. Although our current analysis is limited by medical CT nominal voxel dimensions and grain width issues not encountered in our previous study, micro CT, which can attain resolution to 4 microns shows great promise as a means of bypassing these issues. However, in exchange for the gain in image quality there are negative aspects such as calibration difficulties to absolute as well as intra-machine, measurement repeatability difficulties, and the largest hurdle that current machine fixed detector rings are not large enough to accommodate an object the size of a violin. Adaptive geometry micro CT machines are slowly becoming more common which allow passage of violins, and increased usage and demand will likely address the calibration and repeatability issues in the near future. Another option is synchrotron radiation micro CT which is capable of producing 1.5 and smaller micron voxels although very high cost will limit its role.

In conclusion, based on the well-understood relationships between density, wood stiffness, and internal damping, our results indicate increasing difficulty in sustaining the notion that the classical Cremonese violin makers such as Stradivari and Guarneri del Gesu had access to wood with significantly different material properties than what contemporaneous could, or modern makers can, access.

## References

[1] Cremer L (1984) The Physics of the Violin, MIT Press.

[2] Rossing TD, editor (2007) Handbook of Acoustics, Springer.

[3] Barlow CY (1997) Materials Selection for Musical Instruments. Proc Inst Acoust 19 (5) 69–78.

[4] Woodhouse J (2001) Body Vibration of the Violin. What Can a Maker Expect to Control? Catgut Acoust Soc J 4 (5, 2) 43–49.

[5] Panshin AF, de Zeeuw C (1980) Textbook of Wood Technology. Structure, Identification, Properties, and Uses of the Commercial Woods of the United States and Canada, 4th edn, McGraw-Hill, New York.

[6] Olesen PO (1976) The Interrelation between Basic Density and Ring Width of Norway Spruce. Det Forstlige Forsøksvaesen i Danmark 34 (4) 339–359.

[7] Frimbong-Mensah K (1987) Fibre length and basic density variation in the wood of Norway spruce (Picea abies L. Karst.) from northern Norway. Communications of the Norwegian Forest Research Institute 40: 1–25.

[8] Petty JA, Macmillan DC, Steward CM (1990) Variation of density and growth ring width in stems of Sitka and Norway spruce. Forestry 63 (1) 39–49.

[9] Danborg F (1994) Density variation and demarcation of the juvenile wood in Norway spruce. Danish Forest and Landscape Research Institute, Forskningserien 10: 1–78.

[10] Saranpää P(2003) Wood density and growth. In: Barnett, J.R. & Jeronimidis, G. (eds.). Wood quality and its biological basis. Blackwell Publishing & CRC Press, Biological Sciences Series, Bodmin, Great Britain: 87–117.

[11] Bucor V (2006) Acoustics of Wood, Springer, New York.

[12] Stoel B, Borman T (2008) A Comparison of Wood Density between Classical Cremonese and Modern Violins. PLoS ONE 7 10.1371/journal.pone.0002554 PMC2438473 18596937

[13] Norimoto M, Gril J, Sasaki T, Rowell RM (1988) Improvement of acoustical properties of wood through chemical modifications. Proc Conf Comportement Mecanique du Bois, Bordeaux, 9-9 June: 37–44.

[14] Yano H, Minato K (1992) Improvement of the acoustic and hygroscopic properties of wood by a chemical treatment and applications to the violin parts. J Acoust Soc Am 92: 1222–1227.

[15] Yano H, Kajita H, Minato K (1994) Chemical Treatment of Wood for Musical Instruments. J Acoust Soc Am 96: 3380–3391.

[16] Barlow CY, Woodhouse J (1992) Bordered pits in spruce from old Italian violins. J Microsc 160 (2) 203–211.

[17] Mork E (1928) Qualität des Fichtenholzes unterbesonderer Rucksichtnahme auf Schleif - und Papierholz. Pap Fabrik 26: 741–747.

[18] Jozsa L, Richards J, Johnson S (2011) Calibration of Forintek's Direct Reading Densitometer. CFS Contract Report No. 55-12-001.

[19] Polge H (1978) Fifteen Years of Wood Radiation Densitometry. Wood Sci Technol 12: 187–196.

[20] Koubaa A, Zhang Tony S, Makni S (2002) Defining the Transition from Early Wood to Latewood in Black Spruce Based on Intra-ring Wood Density Profiles from X-ray Densitometry. Ann For Sci 59: 511–518.

[21] Niklas K (1993) Influence of Tissue Density-specific Mechanical Properties on the Scaling of Plant Height. Annals of Botany 72: 173–179.

[22] Ashby M, Gibson L, Wegst U, Olive R (1995) The Mechanical Properties of Natural Materials. 1.Material Property Charts. Proc R Soc Lond A 450: 123–140.

[23] Detloff JA (1985) Statistical Relationships Between Acoustic Parameters of Violin Tonewoods. J Catgut Acoust Soc Number 43: 13–15.

[24] Niklas K, Spatz H (2010) Worldwide Correlations of Mechanical Properties and Green Wood Density. American Journal of Botany 97: 1587–1594. 21616793 10.3732/ajb.1000150

[25] Tjoelker MG, Boratynski A, Bugala V(2007) Biology and Ecology of Norway Spruce. Springer, New York.

[26] Barlow CY (1997) Materials Selection for Musical Instruments,. Proc of the Institute of Acoustics 19: 69–78.

[27] Sjostrom E (1993) Wood Chemistry, Fundamentals and Applications, Academic Press, London.

[28] Walker JCF (1993) Primary Wood Processing, Principles and Practice, Chapman & Hill, London. 595: 74–77.

[29] Gortner A (1938) Analysis of Glacial and Preglacial Woods. J Am Chem Soc 60: 2509–2511.

[30] Pishik I, Fefilon V, Burkovskaya V (2011) Chemical Composition and Chemical Properties of New and Old Wood. Lesnoi J 14: 89–93.

